# Simulation Study and Optimization Strategies for Vacuum Infusion of GFRP Hoses Based on Resin Time-Viscosity Variables

**DOI:** 10.3390/polym16101328

**Published:** 2024-05-09

**Authors:** Yue Jiang, Jiazhong Xu, Meijun Liu, Tianyu Fu

**Affiliations:** 1 School of Mechanical Power Engineering, Harbin University of Science and Technology, Harbin 150080, China; 1920510098@stu.hrbust.edu.cn; 2 School of Automation, Harbin University of Science and Technology, Harbin 150080, China; liumeijun@hrbust.edu.cn; 3 Department of Intelligent Equipment, Changzhou College of Information Technology, Changzhou 213164, China; futianyu@ccit.js.cn

**Keywords:** GFRP, VARTM, finite element analysis, infusion process, resin viscosity

## Abstract

During the infusion process of a glass-fiber-reinforced thermosetting composite hose, the viscosity of its resin matrix undergoes temporal variations. Consequently, if the impact of resin viscosity changes over time on the internal resin fluidity is not considered during the infusion process, this may result in the incomplete impregnation of the hose, characterized by the presence of numerous voids. This phenomenon adversely affects the quality of the pipe’s curing and forming process. Therefore, based on the characteristic variations in resin viscosity, this paper considers the changes in fluidity caused by the resin’s temporal evolution within the material. We establish a finite element simulation model to calculate and analyze the overall infusion effects of resin viscosity changes during the hose infusion process. Furthermore, based on the predicted analysis, a variable parameter infusion strategy is proposed to increase resin impregnation in the hose, thereby reducing internal void content and subsequently improving the quality of material curing and forming.

## 1. Introduction

Glass-fiber-reinforced plastic (GFRP) pipelines possess excellent corrosion resistance, lightweight properties, high specific strength, rational mechanical performance, low thermal stress, good electrical insulation, and anti-contamination characteristics [1,2,3]. They can also be seamlessly integrated with various materials through multiple connection methods, offering strong design versatility and compatibility with UV curing [4,5,6]. Consequently, GFRP finds extensive applications in pressure pipelines within oil fields, chemical pipelines, drainage systems, mechanical circulating water pipelines in power plants, and pipelines handling corrosive gases [7].

Vacuum-assisted resin transfer molding (VARTM) utilizes negative pressure from a vacuum to facilitate resin impregnation into fiber fabrics [8]. In comparison to conventional resin transfer molding (RTM), the vacuum properties in the pre-filled region of VARTM enhance the ratio of fiber to resin after impregnation, effectively reducing porosity and increasing the strength of the product after curing [9]. Additionally, VARTM improves the wettability of fibers during the molding process, resulting in a more seamless interface between resin and fibers, thereby enhancing the overall quality of the product. Consequently, scholarly interest in research on the VARTM process is gradually increasing.

Shevtsov S. et al. [10] present a method for deciding on the rational design of a process layout. And the effectiveness of the proposed method is demonstrated by the example of vacuum infusion of a 3D thin-walled structure of complex geometry. Furthermore, Ricciardi et al. [11] developed a novel pulse perfusion technique for the preparation of fiber-reinforced thermosetting matrix composites. The bending modulus and bending strength of the composites that were prepared by this method were increased by 9% and 24% on average, as compared with the traditional preparation methods. By studying the effect of time and the temperature gradients of the resin perfusion, Maung et al. [12] optimized the perfusion time for large composite materials. They effectively solved problems such as thermal damage during the perfusion of large composite materials. Yalcinkaya et al. [13] compared experimentally the filling time of the vacuum injection molding and resin transfer molding, used the model to conduct compaction and permeability characterization experiments, and applied the results to the simplified vacuum perfusion model. This procedure was more intuitive than the traditional fully coupled model that can be found in the literature. The filling time of the vacuum injection was 31% shorter than that of resin transfer molding. Zhai et al. [14] conducted numerical simulations for the vacuum entry of wind turbine blade roots. They obtained corresponding resin flow patterns under different pipeline arrangements and compared the results with experimental data. The results showed that numerical simulations could predict the risks well during blade perfusion. Based on the analysis of the compacting properties of resin performances and the impregnation of fiber bundles, Yang et al. [15] established a resin flow governing equation for vacuum-assisted resin injection. The authors used an innovative and alternative method to calculate the resin mass changes that were caused by fiber bundle infiltration and prefabricated deformation. They analyzed the influence of deformation and the unsaturated effects of the prefabricated parts in mold filling.

While scholars have conducted extensive research on the molding process and process pressure of VARTM, numerous challenges still exist in the infusion process of GFRP hoses. To enhance the convenience of storing and transporting GFRP hoses, a certain amount of thickening agent is added to the resin matrix, with the intention of reducing the fluidity of the resin to meet transportation and storage standards after the infusion process. Therefore, during the hose infusion process, there is a significant variation in resin viscosity and fluidity over time, especially for GFRP pipe-shaped pre-impregnated materials with large lengths and diameters. The large size of the pre-impregnated material and the extended infusion time, coupled with pronounced changes in resin viscosity, result in the resin being unable to reach all areas of the fiber fabric. This can lead to incomplete impregnation or the occurrence of dry spots even after infusion, significantly impacting the subsequent performance of the pipeline.

This paper focuses on the VARTM process for large-diameter GFRP hose and analyzes the factors influencing its infusion quality. Employing finite element analysis, and based on the characteristic variations in resin viscosity, the study considers the changes in fluidity caused by the resin’s temporal evolution within the material. It establishes a finite element simulation model for the folded configuration of large-diameter GFRP pipe, enabling the rational prediction of the infusion process under different process parameters. We successfully calculated the impact of resin viscosity changes on the infusion pressure and overall infusion effects during the hose infusion process. Based on the observed patterns and infusion results, a reasonable variable parameter infusion strategy is proposed. The effectiveness of infusion strategies is quantitatively assessed through injection performance parameters. The study suggests rational parameters for the infusion molding of large-diameter GFRP pre-impregnated hoses, aiming to enhance the quality of material curing and forming.

## 2. Finite Element Model Building

### 2.1. Theory of Infusion

Based on the continuity equation (Equation (1)) and Darcy’s law (Equation (2)) for the steady flow of a single-phase liquid, and by assuming that the fluid and the porous media preform (composed of reinforced fibers) are both incompressible substances, a basic differential equation (also known as the Laplace equation (Equation (3))) describing the flow of the fluid in the preform has been deduced [16].

(1) $$\frac{\partial \left(\rho \upsilon_{x}\right)}{\partial x}+\frac{\partial \left(\rho \upsilon_{y}\right)}{\partial x}+\frac{\partial \left(\rho \upsilon_{z}\right)}{\partial x}=0$$

(2) $$\upsilon_{x}=-\frac{k\partial \rho }{\mu \partial x},\;\upsilon_{y}=-\frac{k\partial \rho }{\mu \partial y},\;\upsilon_{z}=-\frac{k\partial \rho }{\mu \partial z}$$

(3) $$\frac{\partial^{2}p}{\partial x^{2}}+\frac{\partial^{2}p}{\partial y^{2}}+\frac{\partial^{2}p}{\partial z^{2}}=0$$

where ρ is the fluid density; μ is the fluid viscosity; k is the permeability of the preformed body; and $\upsilon_{x}$, $\upsilon_{y}$, and $\upsilon_{z}$ are the x, y, z parts of the fluid velocity. In addition, ρ, μ, and k are constants. According to the Darcy equation, the approximate permeability expressions (4)–(7) (in 3D coordinates) have been derived and established (Equations (4), (5), (6), and (7), respectively).

(4) $$\left[\begin{array}{c} \upsilon_{x}\\ \upsilon_{y}\\ \upsilon_{z} \end{array}\right]=-\frac{1}{\mu }\left[\begin{array}{ccc} k_{xx} & k_{xy} & k_{xz}\\ k_{xy} & k_{yy} & k_{yz}\\ k_{xz} & k_{yz} & k_{zz} \end{array}\right]\left[\begin{array}{c} \frac{\partial \rho }{\partial x}\\ \frac{\partial \rho }{\partial y}\\ \frac{\partial \rho }{\partial z} \end{array}\right]$$

(5) $$K_{x}=\frac{\eta a^{2}\left(1-\varphi_{f}\right)}{6\Delta pt}\left[2{\left(\frac{x}{a}\right)}^{3}-{\left(\frac{x}{a}\right)}^{2}+1\right]$$

(6) $$K_{y}=\frac{\eta b^{2}\left(1-\varphi_{f}\right)}{6\Delta pt}\left[2{\left(\frac{y}{b}\right)}^{3}-{\left(\frac{y}{b}\right)}^{2}+1\right]$$

(7) $$K_{z}=\frac{\eta c^{2}\left(1-\varphi_{f}\right)}{6\Delta pt}\left[2{\left(\frac{z}{c}\right)}^{3}-{\left(\frac{z}{c}\right)}^{2}+1\right]$$

where Δp is the pressure difference between the injection port and the flow front, $\varphi_{f}$ is the percentage of fiber volume, and x, y, z is the position coordinate of the flow front. In addition, a, b, c is the spindle of the imaginary semi-ellipsoid injection port.

### 2.2. Geometric Structural Model

For the vacuum perfusion process in the production of a large diameter tubular prepreg, the resin vacuum perfusion is usually conducted in the folded state of the tubular dry material. Before curing, the inside of the tube is inflated, and the pressure makes the tube wall expand to form a round tube. It is therefore necessary to model the large diameter tubular folded state in the study of the perfusion process.

**Figure 1:**
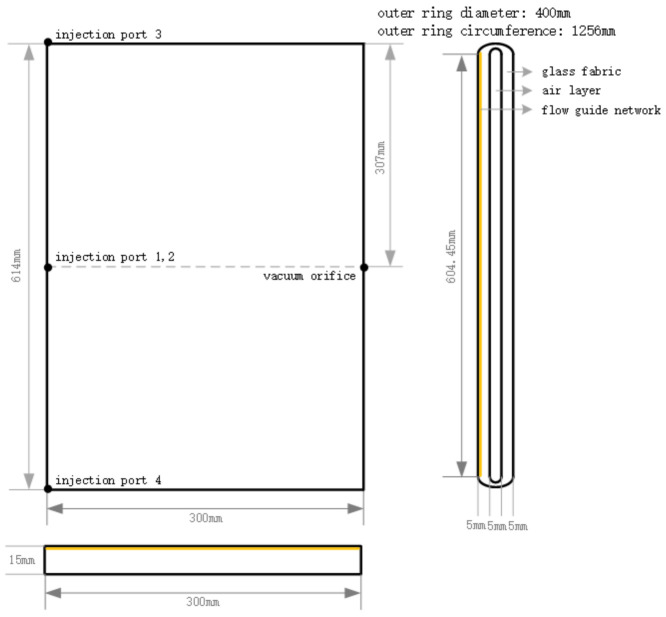
Folding model size of the large-sized GFRP hose.

**Figure 2:**
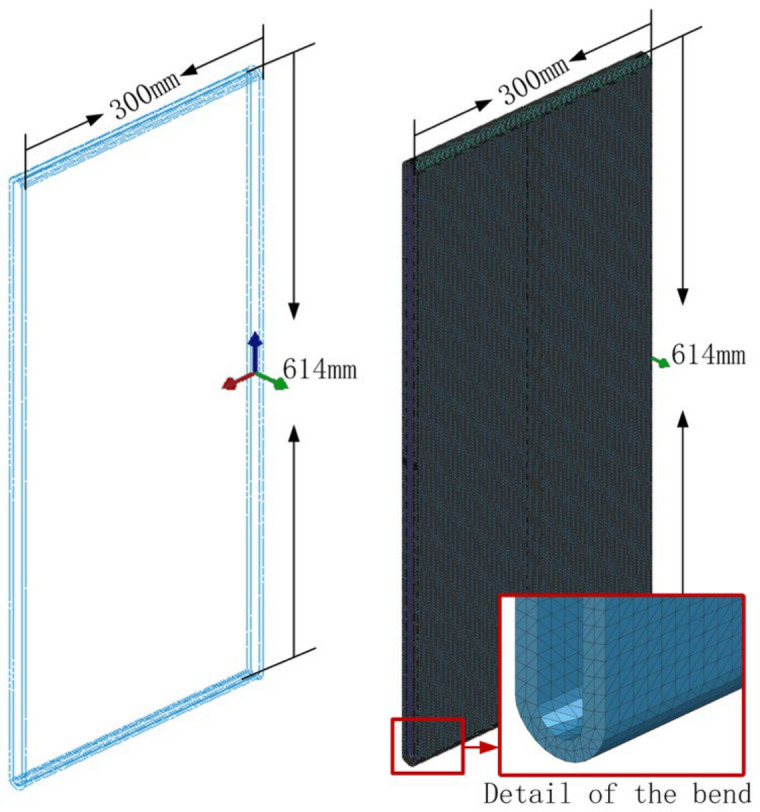
Folding geometry model and meshing of large FRP hose.

To reduce the size of the model and the number of simulations, a section of the hose was selected as the research object, and it was assumed that the material thickness did not change during the perfusion process. Unlike in geometrical modeling, a hose with a diameter of 400 mm was used as an example. Also, modeling software (PAM-Composite 2018) was used to establish the geometric model; the specific model size is shown in Figure 1. The model generally adopts a quadrilateral mesh. Due to the large geometrical size span between the folded and flat parts on the sides of the GFRP dry material hose, special chamfering had to be performed on the folded parts to set the grid division. This was performed to improve the overall grid quality and simulation speed. The model is divided into 812,261 3D meshes. The mesh details and the grid status, including intricate details at the folded section, are illustrated in Figure 2.

### 2.3. Parameter Settings

#### 2.3.1. Resin Viscosity

For the vacuum perfusion of the large-diameter GFRP prepreg hose, and in order to facilitate the resin infiltration of the glass fiber cloth and later prepreg the hose for storage, thickening agents and additives are usually added to the resin. The viscosity of the resin is therefore low in the initial perfusion and infiltration process and high at the end of the perfusion storage stage. We considered the trend in resin viscosity over time, both experimentally and theoretically, to assure the high accuracy of the theoretical calculations in the present study.

**Figure 3:**
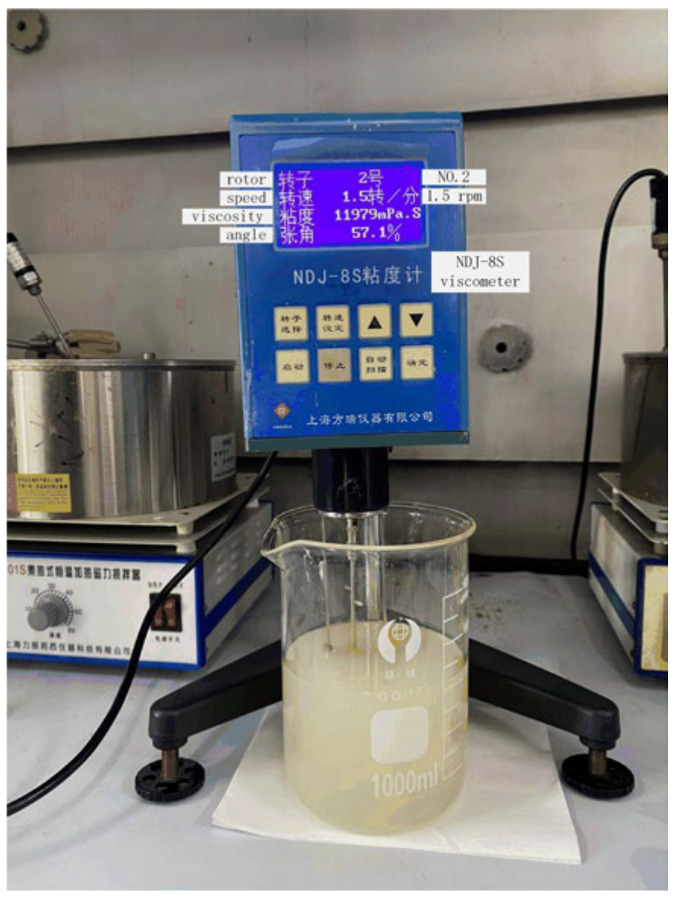
Experimental resin viscosity measurement.

**Figure 4:**
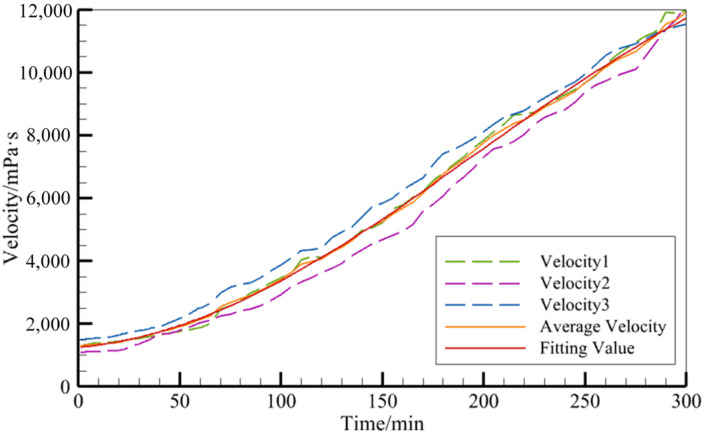
The resin viscosity curve.

At a temperature of 21.2 °C, the resin, thickener, and thickening agent were mixed according to the mass ratio of 100:2:1, and the viscosity changes over time were measured with a rotary viscometer (after that, the mixture was homogeneous). The experimental procedure is depicted in Figure 3. This was repeated three times, after which the resin viscosity variation curve was obtained, as shown by the broken line in Figure 4.

An NDJ-8S rotary viscometer from Shanghai Fangrui Instrument Co., Ltd., Shanghai, China, was used for these experiments. The nr. 2 rotor was selected according to the initial viscosity estimation of the resin. Also, the rotational speed was adjusted according to the percentage of the angle displayed by the instrument in real time, so as to ensure safe and accurate measurements during the experiment.

A variety of fitting methods were used for the regression of the viscosity vs. time function, $\mu \left(t\right)$. Based on the experimental data, the fitting coefficients were all selected in the 95% confidence range. By comparing the quality of the fit (i.e., the R^2^ value) and the root mean square error (RMSE) of the various fitting methods, the principle is to obtain a value as close as possible to R^2^ = 1 and a small RMSE value. This gives a good fitting effect. The polynomial fitting method gave the best fitting result in this study and was therefore selected as the best method to use (as demonstrated by the red solid line in Figure 4). The expression for this method is shown in Equation (8).

(8) μ(t)=7433−6181cos(0.007343x)+782.7sin(0.007343x)

#### 2.3.2. Parameter Settings for Glass Fiber Fabrics

The parameters of the glass fiber fabric were set as shown in Table 1. The diameter of the hose was set to 6 mm in these simulations [17].

## 3. Experimental Verification and Method Deign

### 3.1. Experimental Verification

**Figure 5:**
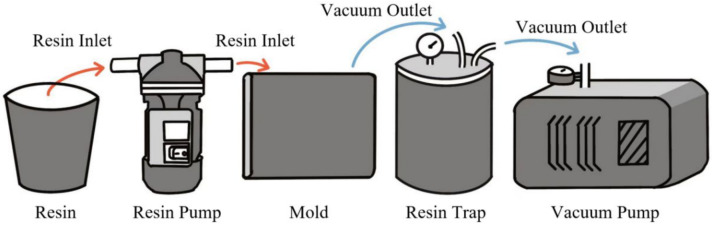
Schematic diagram of the perfusion experiment.

**Figure 6:**
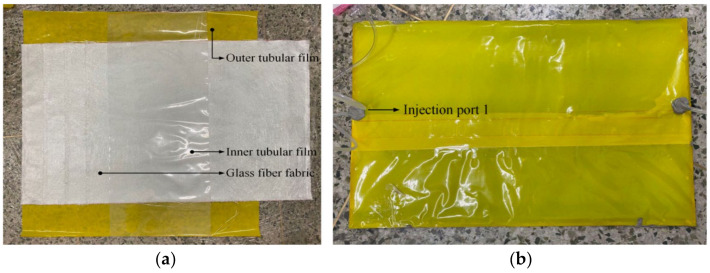
Experimental process diagram. (**a**) Stacking diagram. (**b**) Perfusion process diagram.

Firstly, a tubular dry material was handmade, and its lamination structure consisted of a barrel inner membrane, a glass-fiber-reinforced layer, an impermeable membrane, and an outer membrane. It had the same geometric size as the one used for the theoretical simulations. The links between the experimental equipment are shown in Figure 5. However, the flow guide network was not involved. The upper center point was used to inject the resin, and the constant perfusion pressure was set to and controlled at 3 bar. The perfusion experiment was conducted to observe the resin flow front and the effect of the infiltration on three occasions during the perfusion process. The experimental process is shown in Figure 6.

**Figure 7:**
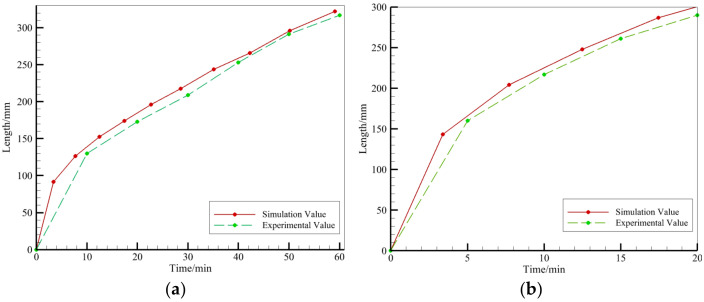
Comparison of resin flow fronts during perfusion processes. (**a**) Pipe ring direction. (**b**) Radial direction.

It was experimentally observed that the resin front reached 300 mm in the pipe diameter direction after 20 min, while it took 19.17 min according to the simulation predictions. Furthermore, the width of the annular resin-infiltrated part was observed experimentally to reach 234 mm after 30 min, while it reached 226.2 mm according to the theoretical simulations. Also, the width of the annular resin-infiltrated part of the tube reached 298 mm after a duration of 50 min in the experiment, and the corresponding simulated width was 286.8 mm. As shown in Figure 7, the calculated predictions were consistent with the experimental results for the single injection nozzle perfusion process, which speaks in favor of the usage of theoretical simulations in forthcoming studies of the vacuum perfusion process of large diameter tubular prepregs. It was calculated that, compared with the experimental results, the trend of the simulation prediction results is the same as the actual detection results, in which the root mean square error of the annular flow front is 9.1 mm, and the root mean square error of the pipe radial flow front is 7.1 mm. For the large-size perfusion target, the annular and radial errors are 1% and 2% of the annular radial dimensions in the plane, respectively, and so the prediction errors are small, which proves that the simulation predicts the accuracy.

### 3.2. Infusion Method Design

The most commonly used perfusion method for the vacuum perfusion processes is a combination of bit perfusion, line perfusion, peripheral perfusion, and mixed perfusion, which are usually combined with a diversion tube and a diversion network. Since the perfusion of a large diameter pipe is conducted in the folded state of the dry material pipe, the folded part at each end produces a large resistance to the resin flow. The two perfusion modes were therefore both considered in the present study. Firstly, natural perfusion without any diversion net was considered and, secondly, auxiliary perfusion with a diversion net was considered.

In the design of the injection port, the vacuum mouth usually needs to consider the following circumstances. If the quantity and location of the injection ports and air vents are different, the resin flow mode and the time necessary to fill the whole product are also different. In general, the more injection ports, the shorter the time it takes to fill the whole product, and the efficiency of the perfusion is also improved. The suction port is designed so that the resin can penetrate the tubular dry material and permeate the fabric completely. The vacuum port should be placed in the area furthest away from the injection port. Furthermore, multiple injection ports and vacuum ports should be designed according to the different shapes of the different areas of the shell. To eliminate the risk of reduced quality, the injection port should be set in the position where quality defects may occur.

**Figure 8:**
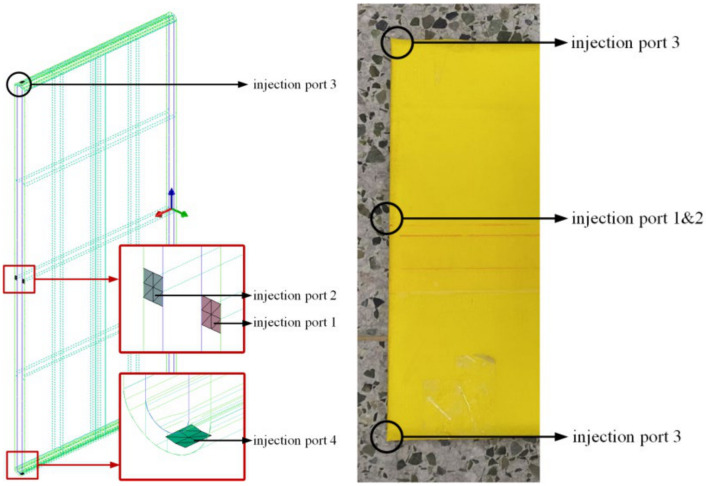
Schematic diagram of the perfusion position.

Based on the present perfusion experience, four injection positions were considered in the present study (Figure 8). With an arrangement including at least one injection port perfusion in combination with at most four injection port perfusions simultaneously, the corresponding perfusion strategy was studied.

## 4. Study and Analysis of the Perfusion Strategy

### 4.1. Perfusion without Any Diversion Network

According to the above-presented finite element analysis model and combined with the comparative study of the experimental results, it has been proven that the establishment of the vacuum perfusion model of the large diameter pipeline was relatively accurate in the present study. Therefore, this model can be used to effectively study the perfusion process and to reduce test costs and research time.

At first, no diversion network was used for the perfusion. When considering the perfusion rule of the model structure, two injection ports and a maximum of four injection ports were preferably used in the present study. Constant pressure perfusion was used in all experiments, and the injection pressure, $P_{inj}$, was set to 3 bar. The design of the perfusion process and the experimental results are shown in Table 2.

The experimental results show that the four simultaneous injection ports, namely the upper and lower center injection ports and the left- and right-side injection ports, finally completed 68.42% of the total perfusion volume. Due to the low completion rate of infusion, the hose cannot be used in engineering applications. Thus, the above four perfusion strategies without any diversion network could not achieve the purpose of the perfusion.

### 4.2. Auxiliary Perfusion of the Diversion Network

To solve the problem of incomplete perfusion and to facilitate the flow of the resin, the following experiment was conducted by positioning a single-layer diversion network in the pipe. As was the case above, constant pressure perfusion was used in this experiment, and the injection pressure, $P_{inj}$, was set to 3 bar. The perfusion strategy and experimental results are shown in Table 3.

Two strategies for successful perfusion have been obtained from these experiments, namely (A) the upper surface center point perfusion, and (B) a perfusion at the center of the upper and lower surface (simultaneous). The completion times for perfusion were 1088.35 s and 3020.61 s, respectively.

### 4.3. Analysis of the Infusion Process and Strategies

**Figure 9:**
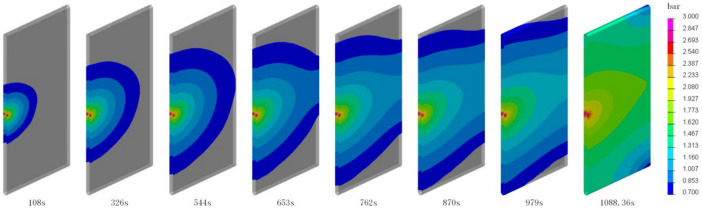
Pressure distribution map during the Strategy A infusion process.

**Figure 10:**
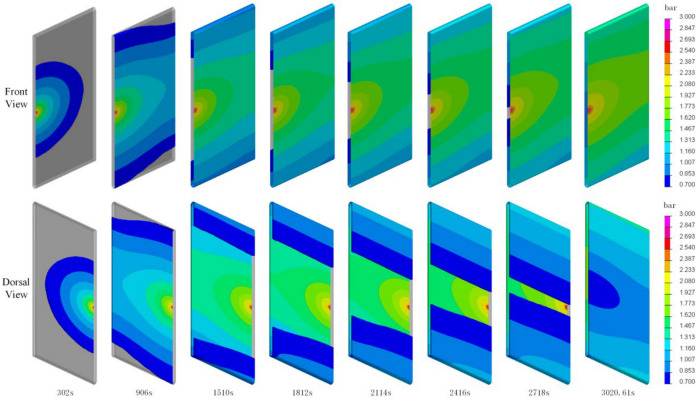
Pressure distribution map during the Strategy B infusion process.

Figure 9 and Figure 10 depict the pressure distribution during the infusion process for two different strategies. As observed in the figures, the pressure decreases gradually from the resin inlet to the advancing front, starting at 3 bar. In the infusion process of Strategy A, resin enters from the central injection points on the upper and lower surfaces, spreading in a semi-circular pattern. It predominantly fills along the central axis, and finally, the upper and lower resin layers meet at the sides, completing the infusion. In the infusion process of Strategy B, resin enters from the central injection point on the upper surface, spreading in a semi-circular pattern towards the sides and then flowing downward to the lower surface. It diffuses from both sides towards the center, and at the lower surface, it converges at the central point away from the injection point on one side. As illustrated in the completed diagram, the point where the resin converges is the location where a vacuum port should be installed during production.

The dimensionless number In [16] was calculated by dividing the injection pressure-to-liquid viscosity ratio with the integral of the cavity filling time (Equation (9)).

(9) $$In=\int_{0}^{inj}\frac{P_{inj}(t)dt}{\mu \left[\alpha (t),T(t)\right]}$$

where $t_{inj}$ is the perfusion time, μ is the resin viscosity, and $P_{inj}$ is the perfusion pressure.

The dimensionless number is independent of the perfusion pressure, perfusion flow, and resin viscosity. It provides an evaluation of the difficulties with liquid injection for porous materials with a given perfusion model geometry, permeability distribution, and injection position.

Regarding the two gluing methods, A and B, the gluing method was changed for the experiment, and the dimensionless number In was calculated and compared with the advantages and disadvantages of the two perfusion strategies. The results are shown in Table 4. The smaller the value of In, the more reasonable the perfusion strategy.

The results showed that the completion time of the constant pressure perfusion was much longer than that of the constant flow perfusion for two kinds of perfusion strategies: resin injection at the center of the upper surface and resin injection at the center of the upper and lower surface. Within the same perfusion strategy, a controlled pressure constant flow perfusion can effectively shorten the completion time of the perfusion without affecting the dimensionless number In.

### 4.4. Mechanical Performance Testing

Utilizing the successful and optimized infusion strategy determined from the previous modeling and analysis, the folded-state tubes were infused accordingly. After curing, five specimens were cut from the pipe wall according to standards. Mechanical testing using a universal testing machine was conducted for three-point bending. The elastic modulus was employed to describe the material’s degree of elastic deformation under stress, with a higher elastic modulus indicating greater resistance to elastic deformation and higher stiffness. The bending strength was used to characterize the material’s tensile strength under bending loads, measuring its resistance to tension during bending. The elastic modulus, flexural strength, and circumferential stiffness were calculated based on the following formulas (Equations (10)–(12)).

(10) $$\sigma_{f}=\frac{3P\cdot l}{2b\cdot h^{2}}$$

In the equation, $\sigma_{f}$ represents the flexural strength, P denotes the failure load, l stands for the span, h represents the specimen thickness, and b denotes the specimen width.

(11) $$E_{f}=\frac{l^{3}\cdot \Delta P}{4b\cdot h^{3}\cdot \Delta S}$$

In the equation, $E_{f}$ represents the flexural modulus, ΔP is the load increment corresponding to the initial linear segment of the load-deflection curve, and ΔS is the deflection increment at the midpoint of the span corresponding to the load increment ΔP.

The circumferential stiffness is a crucial performance metric for assessing the pipe’s resistance to radial deformation under external loads. The fundamental definition of circumferential stiffness is represented by the following equation:

(12) $$S=\frac{EI}{D^{3}}$$

In the equation, S represents the torsional stiffness, E is the circumferential bending modulus of the pipe, I is the circumferential bending moment of inertia per unit length of the pipe wall, t is the total thickness of the pipe wall, and D is the calculated diameter of the pipe.

After calculation, the mechanical properties of the cured pipeline are as shown in Table 5 below, meeting the usage standards.

## 5. Conclusions

1. This paper, through experimentation, acquires the temporal variations in resin viscosity. Considering the changes in fluidity caused by the resin’s temporal evolution within the material, a finite element simulation model is established. This model facilitates the calculation and analysis of the overall infusion effects of resin viscosity changes during the hose infusion process.
2. We conducted experiments on hose infusion using a constant-pressure vacuum infusion method without a flow-guiding network. We extracted the position information of the transverse and radial flow fronts during the infusion process and compared it with simulation results, thus validating the accuracy of the established theoretical model. This provides an intuitive and accurate research methodology for studying VARTM process parameters for hoses.
3. Utilizing a finite element model, the infusion process and outcomes for different process parameters were simulated and predicted. The influence of various vacuum infusion parameters on the infusion results was analyzed. By evaluating infusion strategies based on the dimensionless number In, we proposed an infusion strategy involving simultaneous resin injection at the center of the upper surface or the center of both the upper and lower surfaces. The research found that the infusion method that involves maintaining constant flow by controlling the injection pressure can effectively reduce infusion time and minimize the occurrence of dry spots, achieving high-quality molding for large-diameter pipe-shaped pre-impregnated materials.

## Figures and Tables

**Table 1:** Simulation parameter setting.

Materials	Glass Fiber Fabric	Diversion Medium
$K_{x}/\times 10^{-11}$	43	440
$K_{y}/\times 10^{-11}$	3.7	350
$K_{z}/\times 10^{-11}$	0.65	10
Porosity	0.5	0.8
Thickness (mm)	5	1

**Table 2:** Simulation results of perfusion without diversion network.

NO.		1	2	3	4
Injection condition	Upper central point	Yes	Yes	No	Yes
	Lower central point	No	Yes	No	Yes
	Left side	No	No	Yes	Yes
	Right side	No	No	Yes	Yes
Infusion steady-state result (%)		23.91	56.37	22.68	68.42
Infusion time (s)		360	360	360	360

**Table 3:** Simulation results of perfusion with diversion network.

NO.		5	6	7	8
Injection condition	Upper central point	No	No	Yes	Yes
	Lower central point	No	No	Yes	Yes
	Left side	Yes	Yes	No	Yes
	Right side	No	Yes	No	Yes
Infusion steady-state result (%)		23.91	21.18	45.12	99.59
Infusion time (s)		360	360	360	1088.35

**Table 4:** Simulation results of two perfusion modes, A and B.

Case	Test	Injection Condition	Filling Time (s)	Injectability Number (10^7^)
A	Constant injection pressure	P_inj_ = 3 bar	3022.4	4.7
	Lower central point	Q_inj_ = 3 cm^3^/s	374.6	4.7
B	Left side	P_inj_ = 3 bar	1088.34	3.8
	Right side	Q_inj_ = 3 cm^3^/s	172.91	3.8

**Table 5:** The mechanical properties after curing.

Failure Load (N)	Failure Deflection (mm)	Elastic Modulus (MPa)	Flexural Strength (MPa)	Circumferential Stiffness (N/m^2^)
5984.2452	9.3026772	17592.03	552.5619	4242.083

## Data Availability

All data included in this study are available upon request by contact with the corresponding author.
